# Worldwide Effects of Coronavirus Disease Pandemic on Tuberculosis Services, January–April 2020

**DOI:** 10.3201/eid2611.203163

**Published:** 2020-11

**Authors:** Giovanni Battista Migliori, Pei Min Thong, Onno Akkerman, Jan-Willem Alffenaar, Fernando Álvarez-Navascués, Mourtala Mohamed Assao-Neino, Pascale Valérie Bernard, Joshua Sorba Biala, François-Xavier Blanc, Elena M. Bogorodskaya, Sergey Borisov, Danilo Buonsenso, Marianne Calnan, Paola Francesca Castellotti, Rosella Centis, Jeremiah Muhwa Chakaya, Jin-Gun Cho, Luigi Ruffo Codecasa, Lia D'Ambrosio, Justin Denholm, Martin Enwerem, Maurizio Ferrarese, Tatiana Galvão, Marta García-Clemente, José-María García-García, Gina Gualano, José Antonio Gullón-Blanco, Sandra Inwentarz, Giuseppe Ippolito, Heinke Kunst, Andrei Maryandyshev, Mario Melazzini, Fernanda Carvalho de Queiroz Mello, Marcela Muñoz-Torrico, Patrick Bung Njungfiyini, Domingo Juan Palmero, Fabrizio Palmieri, Pavilio Piccioni, Alberto Piubello, Adrian Rendon, Josefina Sabriá, Matteo Saporiti, Paola Scognamiglio, Samridhi Sharma, Denise Rossato Silva, Mahamadou Bassirou Souleymane, Antonio Spanevello, Eva Tabernero, Marina Tadolini, Michel Eke Tchangou, Alice Boi Yatta Thornton, Simon Tiberi, Zarir F. Udwadia, Giovanni Sotgiu, Catherine Wei Min Ong, Delia Goletti

**Affiliations:** Istituti Clinici Scientifici Maugeri IRCCS, Tradate, Italy (G.B. Migliori, R. Centis, A. Spanevello);; Yong Loo Lin School of Medicine, National University of Singapore, Singapore (P.M. Thong, C.W.M. Ong);; University of Groningen, Groningen, the Netherlands (O. Akkerman);; The University of Sydney, Sydney, New South Wales, Australia (J.-W. Alffenaar, J.-G. Cho);; Westmead Hospital, Sydney (J.-W. Alffenaar, J.-G. Cho);; Hospital Universitario San Agustín, Avilés, Spain (F. Álvarez-Navascués, J.A. Gullón-Blanco);; National Tuberculosis Programme, Niamey, Niger (M.M. Assao-Neino);; Centre Hospitalier Universitaire, Nantes, France (P.V. Bernard, F.-X. Blanc);; Community Health Centre, Tombo, Sierra Leone (J.S. Biala);; Moscow Research and Clinical Center for TB Control, Moscow, Russia (E.M. Bogorodskaya, S. Borisov);; Fondazione Policlinico Universitario A. Gemelli IRCCS, Rome, Italy (D. Buonsenso);; University Research Co. LLC, Manila, the Philippines (M. Calnan);; Niguarda Hospital, Milan, Italy (P.F. Castellotti, L.R. Codecasa, M. Ferrarese, M. Saporiti);; Kenyatta University, Nairobi, Kenya (J.M. Chakaya);; Liverpool School of Tropical Medicine, Liverpool, UK (J.M. Chakaya);; Parramatta Chest Clinic, Parramatta, New South Wales, Australia (J.-G. Cho);; Public Health Consulting Group, Lugano, Switzerland (L. D’Ambrosio);; Melbourne Health Victorian Tuberculosis Program, Melbourne, Victoria, Australia (J. Denholm);; University of Melbourne, Melbourne (J. Denholm);; Amity Health Consortium, Johannesburg, South Africa (M. Enwerem);; Universidade Federal da Bahia, Salvador, Brazil (T. Galvão);; Hospital Universitario Central de Asturias, Oviedo, Spain (M. García-Clemente);; Tuberculosis Research Programme SEPAR, Barcelona, Spain (J.-M. García-García);; National Institute for Infectious Diseases ‘L. Spallanzani’ IRCCS, Rome (G. Gualano, G. Ippolito, F. Palmieri, P. Scognamiglio, D. Goletti);; Instituto Vaccarezza, Buenos Aires, Argentina (S. Inwentarz, D.J. Palmero);; Royal London Hospital of Barts Health National Health Service Trust, London, UK (H. Kunst, S. Tiberi);; Queen Mary University, London (H. Kunst, S. Tiberi);; Northern State Medical University, Arkhangelsk, Russian Federation (A. Maryandyshev);; Istituti Clinici Scientifici Maugeri IRCCS, Pavia, Italy (M. Melazzini);; Instituto de Doenças do Tórax, Universidade Federal do Rio de Janeiro, Rio de Janeiro, Brazil (F.C.Q. Mello);; Instituto Nacional De Enfermedades Respiratorias Ismael Cosio Villegas, Mexico City, Mexico (M. Muñoz-Torrico);; Community Health Center, Hastings, Sierra Leone (P.B. Njungfiyini; M.E. Tchangou);; Amedeo di Savoia Hospital, Turin, Italy (P. Piccioni);; Damien Foundation, Niamey, Niger (A. Piubello, M.B. Souleymane);; University Hospital of Monterrey (Universidad Autonoma de Nuevo Leon), Monterrey, Mexico (A. Rendon);; Hospital Transversal Moises Broggi-HGH, Barcelona (J. Sabriá);; P.D. Hinduja National Hospital and Medical Research Centre, Mumbai, India (S. Sharma, Z.F. Udwadia);; Universidade Federal do Rio Grande do Sul (UFRGS), Porto Alegre, Brazil (D.R. Silva);; University of Insubria, Varese-Como, Italy (A. Spanevello);; Hospital de Cruces, Vizcaya, Spain (E. Tabernero);; Unit of Infectious Diseases Alma Mater Studiorum University of Bologna, Bologna, Italy (M. Tadolini);; Saint John of God Catholic Hospital, Mabesseneh Lunsar, Sierra Leone (A.B.Y. Thornton);; University of Sassari, Sassari, Italy (G. Sotgiu);; National University of Singapore, Singapore (C.W.M. Ong)

**Keywords:** 2019 novel coronavirus disease, coronavirus disease, COVID-19, severe acute respiratory syndrome coronavirus 2, SARS-CoV-2, viruses, respiratory infections, zoonoses, tuberculosis and other mycobacteria, TB, health services

## Abstract

Coronavirus disease has disrupted tuberculosis services globally. Data from 33 centers in 16 countries on 5 continents showed that attendance at tuberculosis centers was lower during the first 4 months of the pandemic in 2020 than for the same period in 2019. Resources are needed to ensure tuberculosis care continuity during the pandemic.

The coronavirus disease (COVID-19) pandemic has affected clinical management of tuberculosis (TB) and TB-related services (*1*,*2*). Reports of the first cohorts of patients with COVID-19 and TB have been recently published (*3*,*4*), although it may be difficult to distinguish which infection occurred first (*5*). The effects of COVID-19 on TB diagnostic and programmatic activities are similar (*1*). Almost every country has national TB programs in place, whereas national programs for COVID-19 are urgently needed (*1*,*2*).

The effect of COVID-19 on TB services is estimated to be dramatic, especially in countries where healthcare staff involved in TB management have been reassigned to the COVID-19 emergency. However, apart from local studies (*6*), a comprehensive, multinational description is needed.

The Global Tuberculosis Network, which conducted this study, collaborates with TB centers from 41 countries (*3*,*4*,*6*,*7*). We studied patient attendance at TB centers in 16 countries and compared the volume of TB-related healthcare activities in the first 4 months of the COVID-19 pandemic, January–April 2020, with that for the same period in 2019.

## The Study

We invited 37 TB centers to participate in the study and collected data from 33 centers located in 16 countries on 5 continents (Appendix Tables 1, 2). The participating centers received ethics clearance according to their respective center regulations (*7*,*8*). Active TB disease and latent TB infection (LTBI) were defined according to international guidelines (*9*,*10*). We recorded numbers of patients with active TB discharged from inpatient care, patients with newly diagnosed cases of active TB, patients with active TB visiting outpatient settings, and new and total outpatient visits for LTBI. We defined use of telehealth services as implementation of directly observed therapy during face-to-face virtual teleconsultations, which were considered to be equivalent to outpatient visits and were counted as such. We did not consider patient contact by telephone and emails to be telehealth. Home visits were considered outpatient visits. We also recorded national lockdown dates. If a country reported results from >1 center, we used the sum of the attendances to generate the graphs. Quantitative variables were summarized with absolute (percentage) frequencies.

Of the 16 countries studied, data were contributed by 4 TB centers each in Italy, Russia, Spain, and Brazil; 3 each in Sierra Leone and Niger; 2 in Mexico; and 1 each in 9 other countries (Appendix Tables 1, 2). Lockdowns were imposed in all countries (Appendix Figures 1, 2). The earliest lockdown start date was February 1, 2020 (Australia); the latest was April 7, 2020 (Singapore). By the end of data collection (April 30), none of the 16 countries had reduced lockdown severity.

Data on new active TB cases were available from 32 of the 33 TB centers. Except for 5 centers (Sydney, New South Wales, Australia; San Fernando, the Philippines; Turin, Italy; Asturias, Spain; and London, UK), which each reported stable numbers or moderate increases, new active TB cases decreased in 27 (84%) of the 32 TB centers in the first 4 months of 2020 relative to the same period in 2019 (Appendix Figure 1).

Information about total outpatient TB visits was available for 29 centers but not from Groningen, the Netherlands; Mexico City, Mexico; Porto Alegre, Brazil, and Nairobi, Kenya. A total of 22 (75%) of 29 TB centers from 14 countries registered decreased outpatient visits during the lockdowns.

Active TB–associated hospital discharges differed in 2020 from 2019. Although data were not available for a few centers (Buenos Aires, Argentina; Nairobi; and the 3 centers in Niger), data for San Fernando, Singapore; Mexico City, Groningen, and London indicated minimal or no increase. Active TB–associated hospital discharges for the remaining 23 (82%) of 28 TB centers were lower during the first 4 months of 2020.

Data for LTBI outpatient visits were available from 16 of the 33 TB centers; 13 (81%) recorded decreased total outpatient visits (all except Hastings, Sierra Leone; Alvorada, Brazil; and Barcelona, Spain) (Appendix Figure 2). Data for newly diagnosed LTBIs were available from 19 of the 33 TB centers. New LTBI outpatient visits at 18 (95%) of 19 TB centers (all except Alvorada) were fewer during the lockdown period (Appendix Figure 2).

During the first 4 months of 2020, telehealth services were used by 7 (21%) of the 33 TB centers. The number of patients using telehealth services was reported by 4 centers: Sydney; Mumbai, India; London; and Arkhangelsk, Russia. Increased use of telehealth services correlated with lockdown implementation; most uses were recorded in April 2020 (Appendix Tables 1, 2).

## Conclusions 

For most TB centers during their respective national lockdowns in the first 4 months of 2020, we found reductions in TB-related hospital discharges, newly diagnosed cases of active TB, total active TB outpatient visits, and new LTBI and LTBI outpatient visits. These results may be explained by a general decrease in the use of health services, including emergency services (*11*). Resources for TB service provision were reassigned to other medical services. Outpatient visit numbers may have decreased because of patients’ fear of exposure to severe acute respiratory syndrome coronavirus 2 (*12*). Access to medical services may have decreased because of interruptions in or difficulty accessing public transportation, although health-related travel was permitted in most countries. In some TB centers (e.g., Mexico City), the hospital patient intake system was modified to support COVID-19 admissions, thus severely hindering TB services. In some centers, screening for LTBI was considered a lower priority than screening for active TB or COVID-19. Because of lockdowns, reactivation of active TB in persons with LTBI who did not receive preventive therapy may be expected, such as in contacts recently exposed to TB or in those who are immunocompromised (*13*,*14*). In England, compared with 2019, TB notifications decreased by 16.5% during April and by 37.3% during May 2020; the LTBI program was paused in response to COVID-19 on March 26 (*15*).

Lockdowns have favored the increased use of telemedicine. Telehealth is a new service offered by TB programs. In TB centers surveyed in Australia, Russia, India, and the United Kingdom, telehealth service use increased in the first 4 months of 2020. 

Although our study cannot comprehensively describe all features of TB management, we found that the COVID-19 pandemic had a substantial impact on TB services worldwide. The main strength of our study is the global coverage from 33 TB centers from 16 countries on 5 continents. Limitations include lack of data from some countries. In 9 of the 16 countries, data were limited to reports from only 1 TB center, which may not have fully represented that nation’s TB healthcare activities. In addition, some TB centers were located in countries with low TB incidence (e.g., Italy). The description of the changes in the TB burden over a few months did not allow for appropriate statistical inferences in these countries with low TB incidence. More information about the medium- and long-term effects of the COVID-19 pandemic on TB services after a specified time from the diagnosis of the first COVID-19 patient in each country is needed.

The COVID-19 pandemic seems to have affected TB services in all 16 countries that provided data. At select TB centers, increased use of telehealth services during the pandemic was recorded. Resources urgently need to be channeled to ensure that TB care continues efficiently despite the ongoing COVID-19 pandemic.

Appendix Additional results for study of worldwide effects of coronavirus disease pandemic on tuberculosis services, January–April 2020.
