# Supplementary material for: Worldwide Effects of Coronavirus Disease Pandemic on Tuberculosis Services, January–April 2020
Source: Emerg Infect Dis. 2020 Nov;26(11):2709–12. doi: 10.3201/eid2611.203163 (PMC7588533; doi:10.3201/eid2611.203163)
Supplement: Appendix — Additional results for study of worldwide effects of coronavirus disease pandemic on tuberculosis services, January–April 2020. [file 20-3163-Techapp-s1.pdf]

Article DOI: https://doi.org/10.3201/eid2611.203163

Worldwide Effects of Coronavirus Disease Pandemic on Tuberculosis Services, January–April 2020

Appendix

Appendix Table 1. Patients with active tuberculosis (TB), active TB outpatient visits, use of telehealth for TB outpatient visits, and dates of lockdowns in participating countries, January-April, 2019, and January-April, 2020*

Country	TB center, City	Use of telehealth	Period	TB cases, Discharged Inpatients N.	New TB cases, outpatients N.	TB cases Outpatient visits (Telehealth numbers in brackets) N.	Lockdown and reopening dates
Asia Pacific							
Australia	Parramatta Chest Clinic, Sydney	Yes.	First 4 months 2019	29	35	1,550	Partial lockdown: February 1, 2020 Full lockdown start: March 20, 2020
			January	6	3	368	
			February	6	8	411	
			March	8	8	375	
			April	9	16	396	
			First 4 months 2020	23	52	1,892	
			January	10	8	490	
			February	2	16	571	
			March	4	6	484 (43)	
			April	7	22	230 (74)	
India	Hinduja Hospital & Research Center, Mumbai	Yes. Started on March 27, 2020	First 4 months 2019	46	NA	5,092	Lockdown start: March 24, 2020 (Ongoing)
			January	9	NA	1200	
			February	12	NA	1343	
			March	7	NA	1100	
			April	18	NA	1449	
			First 4 months 2020	17	NA	1,445	
			January	6	NA	508	
			February	4	NA	488	
			March	5	NA	254 (30)	
			April	2	NA	52 (113)	
Philippines	JBL memorial hospital, San Fernando	No†	First 4 months 2019	131	4	430	Lockdown start: March 9, 2020
			January	34	2	120	
			February	29	1	80	
			March	37	1	110	
			April	31	0	120	
			First 4 months 2020	123	14	143	
			January	36	4	43	
			February	14	3	27	
			March	33	5	62	
			April	40	2	11	
Singapore	National University Hospital, Singapore	Yes. Started in May 2020	First 4 months 2019	33	31	63	Lockdown start: April 7, 2020 Partial reopening: June 2, 2020
			January	11	10	17	
			February	9	5	13	
			March	5	7	19	
			April	8	9	14	
			First 4 months 2020	37	16	74	
			January	4	12	33	

Country	TB center, City	Use of telehealth	Period	TB cases, Discharged Inpatients N.	New TB cases, outpatients N.	TB cases Outpatient visits (Telehealth numbers in brackets) N.	Lockdown and reopening dates
			February	5	1	17	
			March	16	2	16	
			April	12	1	8	
Europe							
France	Nantes University Hospital, Nantes	Yes. Started in April 2020‡	First 4 months 2019	31	35	187	Lockdown start: March 17, 2020 Lockdown end: May 11, 2020
			January	12	8	52	
			February	3	3	51	
			March	4	13	32	
			April	12	11	52	
			First 4 months 2020	27	24	262	
			January	6	7	76	
			February	9	6	68	
			March	8	5	55	
			April	4	6	63	
Italy	National Institute for Infectious Diseases (INMI) 'L. Spallanzani' IRCCS, Rome	No.†	First 4 months 2019	82	113	725	Lockdown start: March 10, 2020 Partial reopening: May 4, 2020
			January	21	30	200	
			February	22	18	152	
			March	17	27	191	
			April	22	38	182	
			First 4 months 2020	69	81	468	
			January	19	24	183	
			February	27	27	191	
			March	20	24	62	
			April	3	6	32	
	Villa Marelli, Milano	No.†	First 4 months 2019	NA	80	757§	
			January	NA	24	188	
			February	NA	19	183	
			March	NA	21	196	
			April	NA	16	190	
			First 4 months 2020	NA	65	673§	
			January	NA	17	202	
			February	NA	13	165	
			March	NA	15	147	
			April	NA	20	159	
	Sant'Orsola-Malpighi Polyclinic, Bologna	No.†	First 4 months 2019	38	34	607§	
			January	6	9	152	
			February	9	5	157	
			March	9	9	164	
			April	14	11	134	
			First 4 months 2020	35	28	411§	
			January	9	10	156	
			February	10	7	148	
			March	12	6	47	
			April	4	5	60	
	Amedeo di Savoia Hospital, Turin	No.	First 4 months 2019	NA	39	438§	
			January	NA	12	120	
			February	NA	6	102	
			March	NA	11	100	
			April	NA	10	116	
			First 4 months 2020	NA	41	542§	
			January	NA	11	125	
			February	NA	10	118	
			March	NA	6	134	
			April	NA	14	165	

Country	TB center, City	Use of telehealth	Period	TB cases, Discharged Inpatients N.	New TB cases, outpatients N.	TB cases Outpatient visits (Telehealth numbers in brackets) N.	Lockdown and reopening dates
	Total number for all centers in the first 4 months of 2019			120	266	2,527	
	Total number for all centers in the first 4 months of 2020			104	215	2,094	
Netherlands	TB center Beatrixoord, UMCG, Haren/ Groningen	Yes.¶	First 4 months 2019	26	37	NA	Lockdown start: March 15, 2020 Partial reopening: June 1, 2020
			January	4	10	NA	
			February	11	6	NA	
			March	9	14	NA	
			April	2	7	NA	
			First 4 months 2020	30	20	NA	
			January	5	6	NA	
			February	9	6	NA	
			March	13	5	NA	
			April	3	3	NA	
Russia	Moscow City Research and Clinical Center for TB control, and 2 hospitals for TB patients, Moscow	No.	First 4 months 2019	3,251	976	298,387	Lockdown start: March 30, 2020 Partial reopening (Moscow): June 9,2020 Partial reopening (Arkhangelsk): June 15, 2020
			January	706	233	69,158	
			February	878	264	75,278	
			March	882	245	75,330	
			April	785	234	78,621	
			First 4 months 2020	2,208	877	267,882#	
			January	566	180	69,346	
			February	667	274	84,213	
			March	604	170	71,225	
			April	371	253	43,098	
	Arkhangelsk Clinical Antituberculosis Dispensary, Arkhangelsk	Yes.	First 4 months 2019	183	83	2,557	
			January	47	22	565 (1)	
			February	44	21	670 (3)	
			March	51	16	688 (4)	
			April	41	24	620 (6)	
			First 4 months 2020	153	64	2,053	
			January	31	10	531 (4)	
			February	38	20	596 (5)	
			March	44	19	576 (5)	
			April	40	15	325 (11)	
	Total number for all centers in the first 4 months of 2019			3,434	1,059	300,944	
	Total number for all centers in the first 4 months of 2020			2,361	941	269,935	
Spain	Hospital Transversal Moises Broggi-HGH, Barcelona	No.	First 4 months 2019	12	21	67	Lockdown start: March 14, 2020 Partial reopening: May 2, 2020
			January	4	7	15	
			February	1	2	15	
			March	2	5	18	
			April	5	7	19	
			First 4 months 2020	7	12	74	
			January	0	1	21	
			February	4	5	21	
			March	2	3	20	
			April	1	3	12	
	SESPA Hospital Universitario Central De Asturias (HUCA), Oviedo	No.	First 4 months 2019	10	15	55	
			January	3	3	9	
			February	3	5	11	
			March	2	3	16	
			April	2	4	19	
			First 4 months 2020	8	15	49	
			January	3	5	10	

Country	TB center, City	Use of telehealth	Period	TB cases, Discharged Inpatients N.	New TB cases, outpatients N.	TB cases Outpatient visits (Telehealth numbers in brackets) N.	Lockdown and reopening dates
			February	4	7	11	
			March	0	2	14	
			April	1	1	14	
	SESPA University Hospital San Agustín, Avilés	No.	First 4 months 2019	3	6	9	
			January	1	2	1	
			February	1	2	3	
			March	0	0	1	
			April	1	2	4	
			First 4 months 2020	2	4	12	
			January	1	1	2	
			February	1	2	7	
			March	0	1	1	
			April	0	0	2	
	Hospital de Cruces, Vizcaya	No.	First 4 months 2019	23	38	73	
			January	3	6	9	
			February	7	14	20	
			March	9	13	15	
			April	4	5	29	
			First 4 months 2020	11	24	71	
			January	1	4	27	
			February	4	9	29	
			March	3	6	9	
			April	3	5	6	
	Total number for all centers in the first 4 months of 2019			48	80	204	
	Total number for all centers in the first 4 months of 2020			28	55	206	
United Kingdom	Barts Health NHS trust, London**	Yes.	First 4 months 2019	28	80	179	Lockdown start: March 23, 2020
			January	7	23	36	
			February	11	20	67	
			March	5	21	40	
			April	5	16	36	
			First 4 months 2020	16	85	177	
			January	2	19	53	
			February	4	22	53	
			March	5	27	28 (17)	
			April	5	17	2 (24)	
North America							
Mexico	Instituto Nacional De Enfermedades Respiratorias Ismael Cosio Villegas, Mexico city	Yes.‡	First 4 months 2019	NA	82	NA	Partial Lockdown: March 23, 2020 Full Lockdown start: March 30, 2020 Reopening: Jun 1, 2020
			January	NA	23	NA	
			February	NA	17	NA	
			March	NA	22	NA	
			April	NA	20	NA	
			First 4 months 2020	NA	45	NA	
			January	NA	14	NA	
			February	NA	15	NA	
			March	NA	16	NA	
			April	NA	0	NA	
	Hospital Universitario de Monterrey, Monterrey	No.	First 4 months 2019	25	76	583	Lockdown start: March 17, 2020
			January	10	11	170	
			February	5	13	155	
			March	5	24	144	
			April	5	28	114	
			First 4 months 2020	272	38	331	

Country	TB center, City	Use of telehealth	Period	TB cases, Discharged Inpatients N.	New TB cases, outpatients N.	TB cases Outpatient visits (Telehealth numbers in brackets) N.	Lockdown and reopening dates
			January	7	17	135	
			February	9	13	126	
			March	130	8	70	
			April	126	0	0	
	Total number for all centers in the first 4 months of 2019			25	158	583	
	Total number for all centers in the first 4 months of 2020			272	83	331	
South America Argentina	Instituto Vaccarezza, Buenos Aires[††]	No. [†]	First 4 months 2019	NA	78	1,182	Lockdown start: March 20, 2020 Reopen: May 8, 2020
			January	NA	21	321	
			February	NA	21	267	
			March	NA	18	281	
			April	NA	18	313	
			First 4 months 2020	NA	76	1,136	
			January	NA	22	287	
			February	NA	24	273	
			March	NA	23	315	
			April	NA	7	261	
Brazil	Hospital de Clínicas de Porto Alegre, Porto Alegre, Rio Grande do Sul	No.	First 4 months 2019	99	68	NA	Lockdown start: March 17, 2020 (ongoing)
			January	33	27	NA	
			February	28	10	NA	
			March	13	8	NA	
			April	25	23	NA	
			First 4 months 2020	76	52	NA	
			January	24	14	NA	
			February	25	9	NA	
			March	15	19	NA	
			April	12	10	NA	
	Alvorada Tuberculosis Outpatient Clinic, Alvorada, Rio Grande do Sul[††]	No.	First 4 months 2019	NA	65	550	
			January	NA	24	135	
			February	NA	16	121	
			March	NA	13	143	
			April	NA	12	151	
			First 4 months 2020	NA	63	574	
			January	NA	25	144	
			February	NA	9	127	
			March	NA	16	148	
			April	NA	13	155	
	Thoracic Diseases Institute, Rio de Janeiro	No.	First 4 months 2019	18	43	592	
			January	6	13	158	
			February	2	10	160	
			March	9	11	137	
			April	1	9	137	
			First 4 months 2020	15	35	642	
			January	1	11	149	
			February	5	7	208	
			March	7	10	218	
			April	2	7	67	
	Hospital Especializado Octávio Mangabeira, Salvador, Bahia	No.	First 4 months 2019	62	161	808	
			January	12	45	229	
			February	15	38	175	
			March	23	41	187	
			April	12	37	217	

Country	TB center, City	Use of telehealth	Period	TB cases, Discharged Inpatients N.	New TB cases, outpatients N.	TB cases Outpatient visits (Telehealth numbers in brackets) N.	Lockdown and reopening dates
			First 4 months 2020	45	118	724	
			January	9	36	214	
			February	11	32	185	
			March	13	44	214	
			April	12	6	111	
	Total number for all centers in the first 4 months of 2019			179	337	1950	
	Total number for all centers in the first 4 months of 2020			136	268	1940	
Africa							
Kenya	Nairobi	No.	First 4 months 2019	NA	4270	NA	Partial lockdown start: March 20, 2020
			January	NA	1087	NA	
			February	NA	1010	NA	
			March	NA	1122	NA	
			April	NA	1051	NA	
			First 4 months 2020	NA	3733	NA	
			January	NA	1104	NA	
			February	NA	1005	NA	
			March	NA	1063	NA	
			April	NA	561	NA	
Niger	National Reference Centre against Tuberculosis and respiratory disease, Niamey Regional Hospital of Maradi, Maradi National Hospital of Zinder, Zinder	No.	First 4 months 2019	NA	704	3,569	Lockdown start: March 19, 2020 Lockdown end: May 31, 2020
			January	NA	185	990	
			February	NA	135	867	
			March	NA	170	763	
			April	NA	214	949	
			First 4 months 2020	NA	594	3,011	
			January	NA	139	865	
			February	NA	200	905	
			March	NA	174	815	
			April	NA	81	426	
Sierra Leone	Tuberculosis Outpatient Unit, Tombo Community Health Centre, Western Rural Area, Sierra Leone	No.	First 4 months 2019	NA	58	224	Lockdown start: April 1, 2020 (ongoing)
			January	NA	16	68	
			February	NA	16	50	
			March	NA	11	74	
			April	NA	15	32	
			First 4 months 2020	NA	40	132	
			January	NA	17	46	
			February	NA	10	50	
			March	NA	8	27	
			April	NA	5	9	
	Hastings Community Health Center, Sierra Leone	No.	First 4 months 2019	50	59	44	
			January	18	21	15	
			February	13	18	14	
			March	15	16	13	
			April	4	4	2	
			First 4 months 2020	0	55	42	
			January	0	12	10	
			February	0	14	10	
			March	0	17	12	
			April	0	12	10	
	Saint John of God Catholic Hospital, Mabesseneh Lunsar, Sierra Leone	No.	First 4 months 2019	NA	38	158	
			January	NA	10	43	
			February	NA	10	44	
			March	NA	11	43	
			April	NA	7	28	

Country	TB center, City	Use of telehealth	Period	TB cases, Discharged Inpatients N.	New TB cases, outpatients N.	TB cases Outpatient visits (Telehealth numbers in brackets) N.	Lockdown and reopening dates
			First 4 months 2020	NA	19	124	
			January	NA	9	37	
			February	NA	3	33	
			March	NA	3	35	
			April	NA	4	19	
	Total number for all centers in the first 4 months of 2019			50	155	426	
	Total number for all centers in the first 4 months of 2020			0	114	298	

*NA, not available.
†Some patients were followed-up by phone or video calls, but details are unavailable.
‡Use of telehealth, breakdown unavailable
§Data on outpatient visits includes also those for Nontuberculous Mycobacterial Pulmonary Disease (NTM-PD) cases. Center is unable to differentiate NTM-PD and active TB visits in its informatics system
¶Use of video observed treatment (VOT) for some specific cases, breakdown unavailable.
#Data includes numbers of home visits by doctors or nurses. The numbers of home visits from January to April 2020, in chronological order, are 3282, 3660, 3146 and 9559.
**Only patients under the Infectious Diseases and not Respiratory Clinics were included
††Center is an outpatient clinic.

Appendix Table 2. Patients with latent tuberculosis infection (LTBI) and LTBI outpatient visits in different centers in participating countries, January-April, 2019, and January-April, 2020*

Country	TB center, City	Period	LTBI cases Outpatients (New cases) N.	LTBI screening/ preventive therapy Outpatient visits (Telehealth numbers in brackets) N.
Asia Pacific				
Australia	Parramatta Chest Clinic, Sydney	First 4 months 2019	127	619
		January	18	122
		February	30	146
		March	53	185
		April	26	166
		First 4 months 2020	110	418
		January	21	114
		February	30	127
		March	36	96 (2)
		April	23	47 (32)
India	Hinduja Hospital & Research Center, Mumbai	First 4 months 2019	NA	NA
		January	NA	NA
		February	NA	NA
		March	NA	NA
		April	NA	NA
		First 4 months 2020	NA	NA
		January	NA	NA
		February	NA	NA
		March	NA	NA
		April	NA	NA
Philippines	JBL memorial hospital, San Fernando	First 4 months 2019	NA	NA
		January	NA	NA
		February	NA	NA
		March	NA	NA
		April	NA	NA
		First 4 months 2020	NA	NA
		January	NA	NA
		February	NA	NA

Country	TB center, City	Period	LTBI cases Outpatients (New cases) N.	LTBI screening/ preventive therapy Outpatient visits (Telehealth numbers in brackets) N.
		March	NA	NA
		April	NA	NA
Singapore	National University Hospital, Singapore	First 4 months 2019	14	49
		January	3	13
		February	4	15
		March	0	10
		April	7	11
		First 4 months 2020	7	21
		January	3	9
		February	1	1
		March	1	5
		April	2	6
Europe				
France	Nantes University Hospital, Nantes	First 4 months 2019	53	99
		January	15	27
		February	14	25
		March	12	28
		April	12	19
		First 4 months 2020	38	100
		January	15	28
		February	15	28
		March	7	29
		April	1	15
Italy	National Institute for Infectious Diseases (INMI) ‘L. Spallanzani’ IRCCS, Rome	First 4 months 2019	114	543
		January	29	150
		February	30	114
		March	29	143
		April	26	136
		First 4 months 2020	90	350
		January	30	137
		February	38	143
		March	14	46
		April	8	24
	Villa Marelli, Milano	First 4 months 2019	366†	2,661
		January	78	754
		February	136	644
		March	73	668
		April	79	595
		First 4 months 2020	231†	2,089
		January	66	754
		February	71	644
		March	62	381
		April	32	310
	Sant’Orsola-Malpighi Polyclinic, Bologna	First 4 months 2019	NA	308
		January	NA	97
		February	NA	78
		March	NA	83
		April	NA	50
		First 4 months 2020	NA	162
		January	NA	69
		February	NA	54
		March	NA	32
		April	NA	7
	Amedeo di Savoia Hospital, Turin	First 4 months 2019	88†	550
		January	26	151
		February	18	134
		March	19	119
		April	25	146
		First 4 months 2020	90†	388
		January	33	159
		February	18	138
		March	26	73
		April	13	18

Country	TB center, City	Period	LTBI cases Outpatients (New cases) N.	LTBI screening/ preventive therapy Outpatient visits (Telehealth numbers in brackets) N.
	Total number for all centers in the first 4 months of 2019		568	4,062
	Total number for all centers in the first 4 months of 2020		411	2,989
Netherlands	TB center Beatrixoord, UMCG, Haren/ Groningen	First 4 months 2019	NA	NA
		January	NA	NA
		February	NA	NA
		March	NA	NA
		April	NA	NA
		First 4 months 2020	NA	NA
		January	NA	NA
		February	NA	NA
		March	NA	NA
		April	NA	NA
Russia	Moscow City Research and Clinical Center for TB control, and 2 hospitals for TB patients, Moscow	First 4 months 2019	NA	NA
		January	NA	NA
		February	NA	NA
		March	NA	NA
		April	NA	NA
		First 4 months 2020	NA	NA
		January	NA	NA
		February	NA	NA
		March	NA	NA
		April	NA	NA
	Arkhangelsk Clinical Antituberculosis Dispensary, Arkhangelsk	First 4 months 2019	994	2,321
		January	178	445
		February	277	621
		March	295	670
		April	244	585
		First 4 months 2020	649	1,639
		January	143	523
		February	165	467
		March	210	546
		April	131	103
	Total number for all centers in the first 4 months of 2019		994	2,321
	Total number for all centers in the first 4 months of 2020		649	1,639
Spain	Hospital Transversal Moises Broggi-HGH, Barcelona	First 4 months 2019	28	54
		January	3	8
		February	9	16
		March	11	19
		April	5	11
		First 4 months 2020	31	74
		January	11	21
		February	9	24
		March	7	18
		April	4	11
	SESPA Hospital Universitario Central De Asturias (HUCA), Oviedo	First 4 months 2019	76	NA
		January	14	NA
		February	15	NA
		March	22	NA
		April	25	NA
		First 4 months 2020	61	NA
		January	23	NA
		February	16	NA
		March	11	NA
		April	11	NA
	SESPA Universitary Hospital San Agustín, Avilés	First 4 months 2019	7	10
		January	4	4
		February	1	4
		March	0	0
		April	2	2
		First 4 months 2020	5	5

Country	TB center, City	Period	LTBI cases Outpatients (New cases) N.	LTBI screening/ preventive therapy Outpatient visits (Telehealth numbers in brackets) N.
		January	1	1
		February	2	2
		March	2	2
		April	0	0
	Hospital de Cruces, Vizcaya‡	First 4 months 2019	39	155
		January	6	44
		February	12	32
		March	14	39
		April	7	40
		First 4 months 2020	26	90
		January	12	37
		February	9	30
		March	3	15
		April	2	8
	Total number for all centers in the first 4 months of 2019		150	219
	Total number for all centers in the first 4 months of 2020		123	169
United Kingdom	Barts Health NHS Trust, London§	First 4 months 2019	73	73
		January	14	14
		February	28	28
		March	15	15
		April	16	16
		First 4 months 2020	40	40
		January	16	16
		February	12	12
		March	9	6 (3)
		April	3	0 (3)
North America				
Mexico	Instituto Nacional De Enfermedades Respiratorias Ismael Cosio Villegas, Mexico city	First 4 months 2019	3	NA
		January	2	NA
		February	0	NA
		March	0	NA
		April	1	NA
		First 4 months 2020	5	NA
		January	2	NA
		February	2	NA
		March	1	NA
		April	0	NA
	Hospital Universitario de Monterrey, Monterrey	First 4 months 2019	77	NA
		January	27	NA
		February	12	NA
		March	21	NA
		April	17	NA
		First 4 months 2020	37	NA
		January	18	NA
		February	15	NA
		March	4	NA
		April	0	NA
	Total number for all centers in the first 4 months of 2019		80	NA
	Total number for all centers in the first 4 months of 2020		42	NA
South America				
Argentina	Instituto Vaccarezza, Buenos Aires	First 4 months 2019	166	915
		January	46	242
		February	28	174
		March	33	199
		April	59	300
		First 4 months 2020	158	756
		January	42	199
		February	45	189
		March	59	248
		April	12	120

Country	TB center, City	Period	LTBI cases Outpatients (New cases) N.	LTBI screening/ preventive therapy Outpatient visits (Telehealth numbers in brackets) N.
Brazil	Hospital de Clínicas de Porto Alegre, Porto Alegre, Rio Grande do Sul	First 4 months 2019	NA	NA
		January	NA	NA
		February	NA	NA
		March	NA	NA
		April	NA	NA
		First 4 months 2020	NA	NA
		January	NA	NA
		February	NA	NA
		March	NA	NA
		April	NA	NA
	Alvorada Tuberculosis Outpatient Clinic, Alvorada, Rio Grande do Sul	First 4 months 2019	25	50
		January	5	10
		February	13	26
		March	4	8
		April	3	6
		First 4 months 2020	26	52
		January	6	12
		February	0	0
		March	4	8
		April	16	32
	Thoracic Diseases Institute, Rio de Janeiro	First 4 months 2019	24	173
		January	6	33
		February	7	38
		March	5	47
		April	6	55
		First 4 months 2020	12	143
		January	6	49
		February	3	37
		March	2	36
		April	1	21
	Hospital Especializado Octávio Mangabeira, Salvador, Bahia	First 4 months 2019	NA	NA
		January	NA	NA
		February	NA	NA
		March	NA	NA
		April	NA	NA
		First 4 months 2020	NA	NA
		January	NA	NA
		February	NA	NA
		March	NA	NA
		April	NA	NA
	Total number for all centers in the first 4 months of 2019		49	223
	Total number for all centers in the first 4 months of 2020		38	195
Africa				
Kenya	Nairobi	First 4 months 2019	NA	NA
		January	NA	NA
		February	NA	NA
		March	NA	NA
		April	NA	NA
		First 4 months 2020	NA	NA
		January	NA	NA
		February	NA	NA
		March	NA	NA
		April	NA	NA
Niger	National Reference Centre against Tuberculosis and respiratory disease, Niamey<br>Regional Hospital of Maradi, Maradi<br>National Hospital of Zinder, Zinder	First 4 months 2019	NA	NA
		January	NA	NA
		February	NA	NA
		March	NA	NA
		April	NA	NA
		First 4 months 2020	NA	NA
		January	NA	NA
		February	NA	NA
		March	NA	NA

Country	TB center, City	Period	LTBI cases Outpatients (New cases) N.	LTBI screening/ preventive therapy Outpatient visits (Telehealth numbers in brackets) N.
		April	NA	NA
Sierra Leone	Tuberculosis Outpatient Unit, Tombo Community Health Centre, Western Rural Area, Sierra Leone	First 4 months 2019	NA	NA
		January	NA	NA
		February	NA	NA
		March	NA	NA
		April	NA	NA
		First 4 months 2020	NA	NA
		January	NA	NA
		February	NA	NA
		March	NA	NA
		April	NA	NA
	Hastings Community Health Center, Sierra Leone	First 4 months 2019	20	19
		January	5	5
		February	4	6
		March	8	6
		April	3	2
		First 4 months 2020	6	6
		January	1	1
		February	2	2
		March	1	1
		April	2	2
	Saint John of God Catholic Hospital, Mabesseneh Lunsar, Sierra Leone	First 4 months 2019	2	NA
		January	1	NA
		February	0	NA
		March	0	NA
		April	1	NA
		First 4 months 2020	1	NA
		January	1	NA
		February	0	NA
		March	0	NA
		April	0	NA
	Total number for all centers in the first 4 months of 2019		22	19
	Total number for all centers in the first 4 months of 2020		7	6

*NA, not available.
†Number of new LTBI cases in Milano and Torino are determined based on the number of patients who just started preventive treatment
‡Some patients were followed-up by phone calls.
§Only patients under the Infectious Diseases and not Respiratory Clinics were included

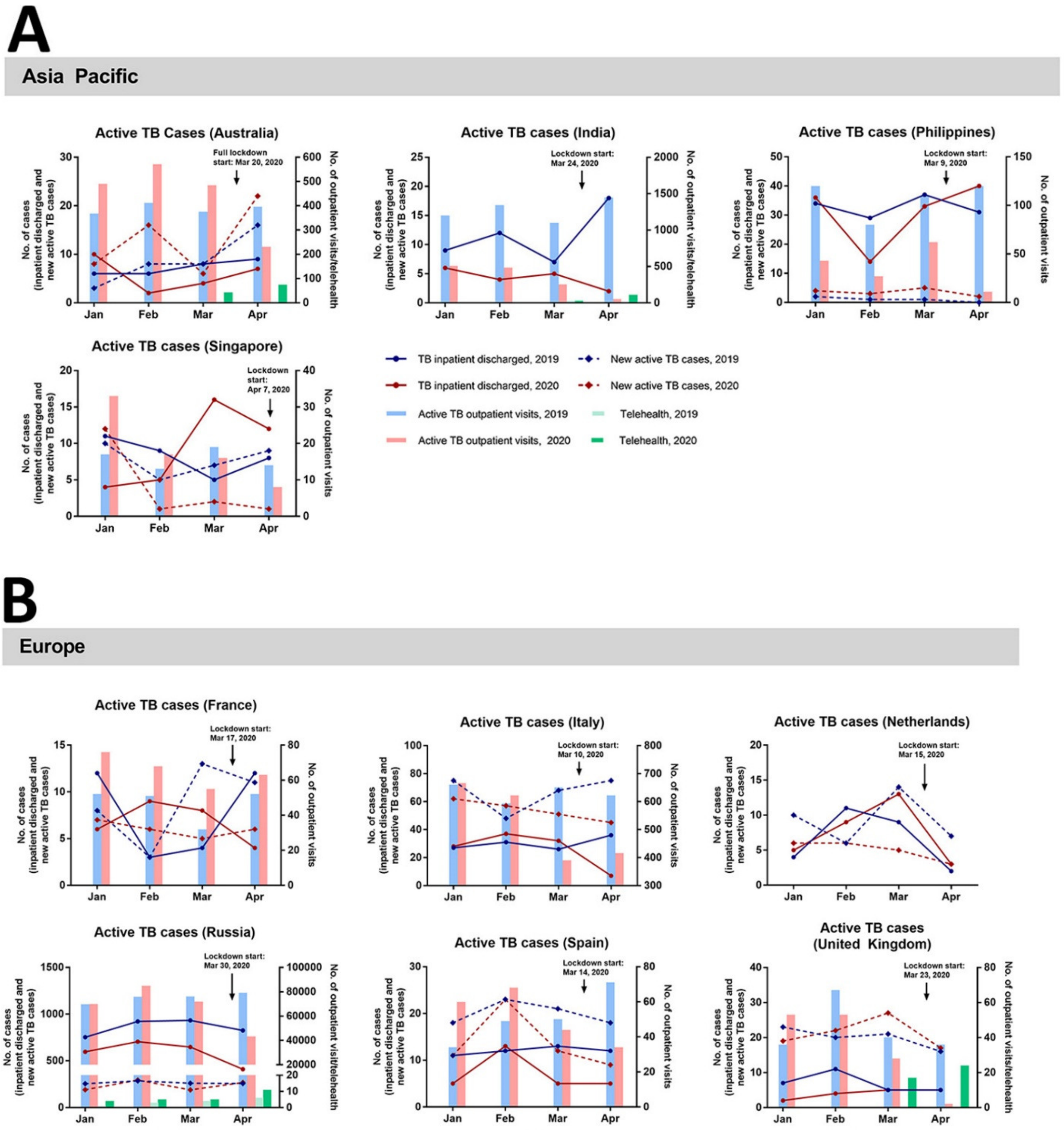
A
Asia Pacific
Active TB Cases (Australia)
Full lockdown start: Mar 20, 2020
Active TB cases (India)
Lockdown start: Mar 24, 2020
Active TB cases (Philippines)
Lockdown start: Mar 9, 2020
Active TB cases (Singapore)
Lockdown start: Apr 7, 2020
No. of cases (inpatient discharged and new active TB cases)
No. of outpatient visits/telehealth
No. of outpatient visits
Jan
Feb
Mar
Apr
TB inpatient discharged, 2019
New active TB cases, 2019
TB inpatient discharged, 2020
New active TB cases, 2020
Active TB outpatient visits, 2019
Telehealth, 2019
Active TB outpatient visits, 2020
Telehealth, 2020
B
Europe
Active TB cases (France)
Lockdown start: Mar 17, 2020
Active TB cases (Italy)
Lockdown start: Mar 10, 2020
Active TB cases (Netherlands)
Lockdown start: Mar 15, 2020
Active TB cases (Russia)
Lockdown start: Mar 30, 2020
Active TB cases (Spain)
Lockdown start: Mar 14, 2020
Active TB cases (United Kingdom)
Lockdown start: Mar 23, 2020

C

North America

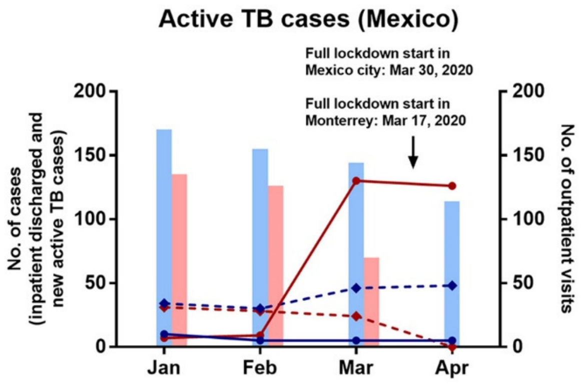

D

South America

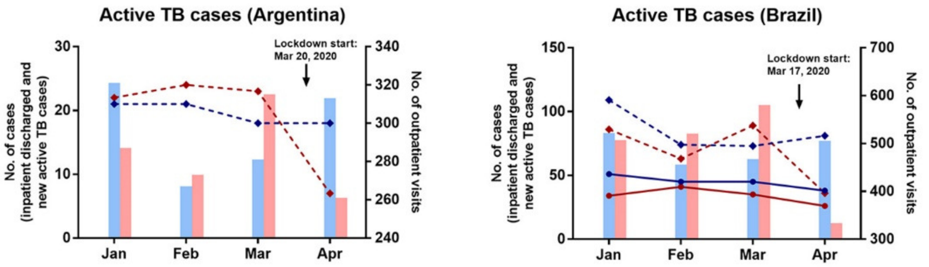

E

Africa

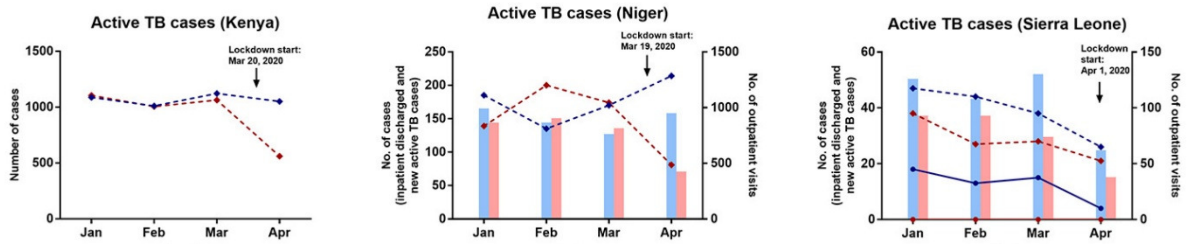

Appendix Figure 1. Impact of COVID-19 on active-TB cases in different TB centers across 16 countries and 5 continents. A) Asia Pacific. B) Europe. C) North America. D) South America. E) Africa.

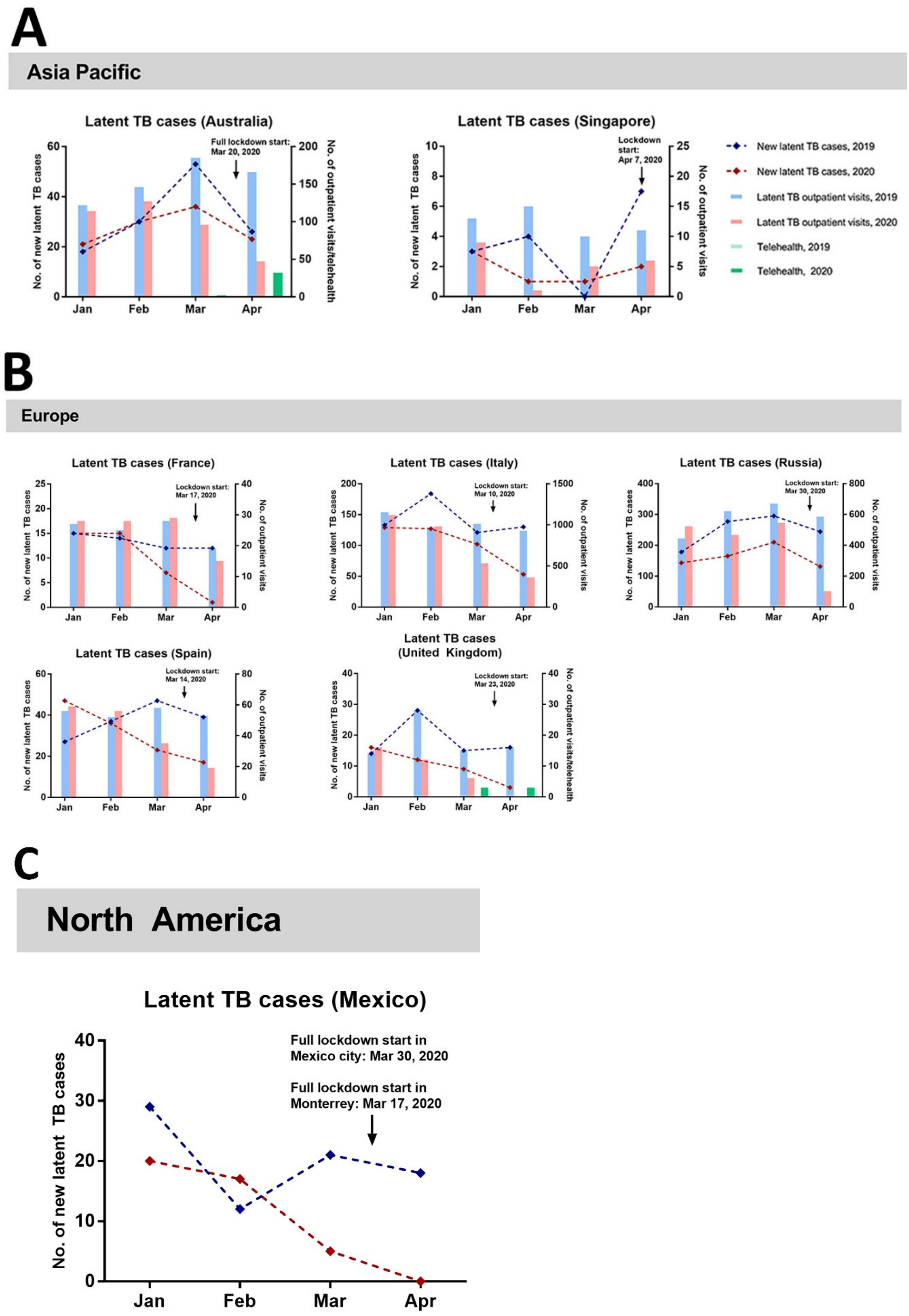
A
Asia Pacific
Latent TB cases (Australia)
Full lockdown start: Mar 20, 2020
No. of new latent TB cases
No. of outpatient visits/telehealth
Jan
Feb
Mar
Apr
Latent TB cases (Singapore)
Lockdown start: Apr 7, 2020
No. of outpatient visits
New latent TB cases, 2019
New latent TB cases, 2020
Latent TB outpatient visits, 2019
Latent TB outpatient visits, 2020
Telehealth, 2019
Telehealth, 2020
B
Europe
Latent TB cases (France)
Lockdown start: Mar 17, 2020
Latent TB cases (Italy)
Lockdown start: Mar 10, 2020
Latent TB cases (Russia)
Lockdown start: Mar 30, 2020
Latent TB cases (Spain)
Lockdown start: Mar 14, 2020
Latent TB cases (United Kingdom)
Lockdown start: Mar 23, 2020
C
North America
Latent TB cases (Mexico)
Full lockdown start in Mexico city: Mar 30, 2020
Full lockdown start in Monterrey: Mar 17, 2020

D

South America

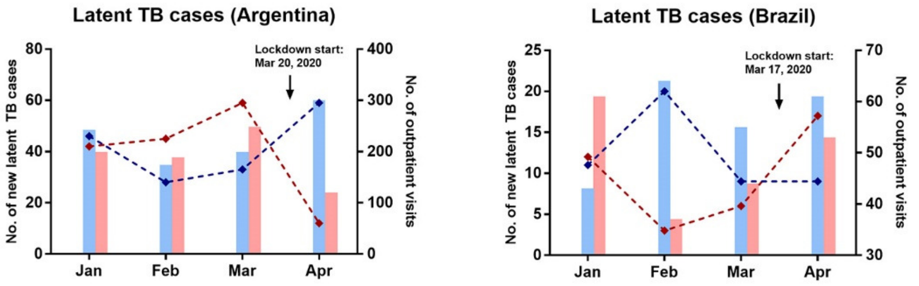

E

Africa

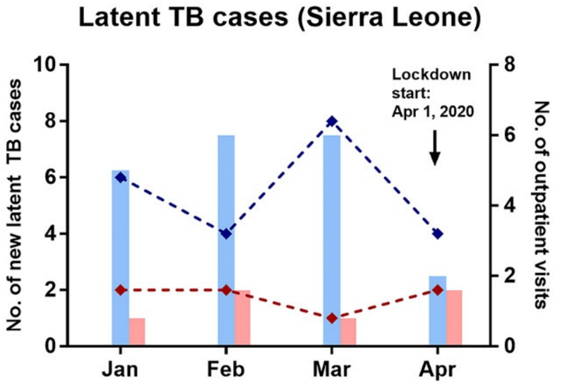

Appendix Figure 2. Impact of COVID-19 on latent TB cases in different TB centers across 11 countries and 5 continents. A) Asia Pacific. B) Europe. C) North America. D) South America. E) Africa.
